# A meta-analysis of the prognostic value of the TyG index in heart failure

**DOI:** 10.3389/fendo.2025.1463647

**Published:** 2025-07-24

**Authors:** Yuqin Cai, Meijie Yang, Shuting Ma, Jinyun Zhang, Bin Huang, Baili Yu

**Affiliations:** ^1^ School of Clinical Medicine, Chengdu University of Traditional Chinese Medicine, Chengdu, Sichuan, China; ^2^ General Medicine Department, Hospital of Chengdu University of Traditional Chinese Medicine, Chengdu, Sichuan, China

**Keywords:** TyG index, heart failure, prognosis, meta-analysis, cardiovascular disease

## Abstract

**Background:**

Heart failure (HF) is a serious cardiovascular disorder with a poor prognosis, which affects the quality of life and survival in patients. The triglyceride-glucose (TyG) index, a new biomarker for insulin resistance (IR) in the body, has attracted widespread attention from researchers investigating cardiovascular disease (CVD). This study was aimed at assessing the prognostic value of the TyG index in HF patients by a meta-analysis, thereby providing clinicians with a new predictive tool.

**Methods:**

PubMed, Cochrane, EMBASE, and Web of Science were searched for studies (from inception to March 2025) on the association of the TyG index with the prognosis of HF. Meta-analysis was conducted using Stata15. Such association was assessed using a random effects model in conjunction with the hazard ratio (HR) and its 95% confidence interval (CI). In addition, subgroup analysis, publication bias analysis, and sensitivity analysis were performed.

**Results:**

Nineteen studies were included with 44275 HF patients. A significant association was found between an increase in the TyG index and an increase in the risk of all-cause death (ACD) in HF patients (HR=1.70, 95% CI: 1.40-2.08, P<0.001). Increased TyG index predicted major adverse cardiovascular events (MACEs) (HR=2.37, 95% CI: 1.80-3.13, P<0.001) and cardiovascular death (CV death) (HR=1.63, 95% CI: 1.01-2.61, P<0.001). Subgroup analysis showed an association of increased TyG index with a poor prognosis regardless of ejection fraction, and the presence or absence of diabetes. Dose-response analysis showed no linear dose-response relationship (DRR) of the index with ACD, MACEs or CV death.

**Conclusion:**

The TyG index is closely associated with the prognosis of HF. Therefore, it can be used as a prognostic tool for the assessment of HF. A high TyG index may indicate a high risk of ACD and CV events. Therefore, monitoring of the TyG index is significant for risk assessment and management of HF patients. Future studies on the use of the TyG index in therapeutic decision-making for HF are needed.

**Systematic review registration:**

https://www.crd.york.ac.uk/PROSPERO/, identifier CRD42024562063.

## 1 Introduction

Heart failure (HF) is a cardiovascular disorder affecting about 64 million people globally. Its prevalence continues to increase annually (1), especially in older adults (2, 3). HF is one of major causes of death related to CVD (4). HF patients have a five-year mortality of about 50% (5). Their prognosis is even worse than that for common cancers (6). Thus, it is crucial to identify HF patients at high risk of poor prognosis. This may help optimize clinical management.

Insulin resistance (IR) is a decreased response of cells in the body to insulin, thereby reducing its function to regulate blood glucose levels. IR has previously been shown to pose a risk of left ventricular (LV) dysfunction and HF (7). It is commonly found in HF patients. It may cause glucose to fail to enter cells efficiently, thereby resulting in increased glucose and triglyceride accumulation (8). IR and triglyceride accumulation may cause metabolic syndrome, which subsequently increases the risk of a poor prognosis for CVD (9–12).

The triglyceride-glucose (TyG) index, as a novel marker of IR, is calculated using the formula: TyG index= Triglyceride (TG) × Glucose (G). The index is considered a simple and reliable measure of IR (13–15). Its positive association with HF has been proven (16). Its prognostic value has also been confirmed in various cardiovascular disorders, such as myocardial infarction (17), hypertension (18), and stroke (19). It may also have a predictive value for HF prognosis, but such value still needs to be further validated. Clinical data has indicated an association of elevated TyG index with a poor prognosis in HF patients (20–22). A meta-analysis (23) showed a high prognostic value of the TyG index in HF patients. Two latest studies (24, 25) showed a negative association of the TyG index level with all-cause death. Therefore, an updated meta-analysis (MA) needs to be done to add further evidence to previous studies. In addition, dose-response analysis of the association between the TyG index and the prognosis in HF patients was performed to provide more references for prognostic assessment of HF patients. This MA systematically explored the predictive potential of the TyG index for prognosis to provide a more reliable tool and rationale for prognostic assessment in clinical settings.

## 2 Methods

### 2.1 Search strategy

This MA was already registered in the PROSPERO (https://www.crd.york.ac.uk/PROSPERO), with an ID of CRD42024562063. It was conducted as per the Preferred Reporting Items for Systematic Reviews and Meta-Analyses (PRISMA) Statement 2020.

Four databases (PubMed, Cochrane, EMBASE, and Web of Science) were systematically searched separately by two reviewers for studies published in English from inception to March 25, 2025, using HF and TyG index (full names inclusive) as search terms. The search strategy is detailed in **Supplementary File 1**.

### 2.2 Inclusion criteria and exclusion criteria

Inclusion criteria according to the PICOS principle (1): Population: HF patients (≥18 years) (2); Exposure: baseline TyG index level (3); Comparator: the highest TyG index vs. the lowest TyG index (4); Outcome: ACD, MACEs, and CV death (5); Study Design: observational studies; and (6) studies providing adjusted HRs with 95% CIs available. Exclusion criteria (1): animal experiments, meta-analyses, reviews, conference abstracts, and case reports (2); duplicate reports (3); studies in languages other than English (4); failure to download full texts.

### 2.3 Data extraction

The studies retrieved were screened separately by two investigators using the predefined inclusion and exclusion criteria. The results from initial search were entered in EndNote 20 (Thomson Reuters, New York, USA). Duplicates were removed. The remaining studies were then subjected to initial screening by title and abstract based on the predefined criteria. Next, the studies that passed initial screening were rescreened by reading their full texts downloaded.

From each included study, two investigators separately extracted the data on (1): general characteristics of the eligible studies: name of the first author, year of publication, country of origin, sample size, and duration of follow-up (2); demographic and clinical characteristics of subjects at baseline: age, gender, diabetes status, left ventricular ejection fraction (LVEF), the highest and lowest TyG index (3); outcomes: adjusted HRs for ACD, CV death, and MACEs, and their 95% confidence intervals (CIs).

### 2.4 Quality assessment

Newcastle-Ottawa Scale (NOS) for cohort study design was used to assess the methodological quality of each selected study (26). NOS consists of three main domains, including selection, comparability, and outcome. This tool was designed and recommended according to the Cochrane Handbook for assessing the quality of observational studies. NOS uses a rating system ranging from 0 to 9 stars to score a study. Studies awarded 7 or more stars are considered high-quality evidence. Studies awarded 4-6 stars are considered moderate-quality evidence. Studies awarded less than 4 stars are considered low-quality evidence. Two authors independently assessed study quality. Discrepancies were resolved by a third author.

### 2.5 Statistical analysis

In this study, the primary outcome measure was ACD, while secondary outcome measures were MACEs and CV death. Adjusted HRs with 95% CIs were summarized to assess the association of the TyG index with the risk of poor prognosis in HF patients. If multivariate analysis used more than one model, then the most adequately adjusted model would be selected. In this MA, the relative risk (RR) was considered an approximate HR. For this index as a categorical variable, the HR was calculated based on comparison of the highest value with the lowest value of this index. In addition, for this index as a continuous variable, the HR reflecting the risk of each unit increase in this index was calculated. The Q test and I^2^ statistics were used for heterogeneity assessment of the studies included. A random effects model was prioritized for merged HRs, because it takes into account both within-study and between-study heterogeneity. A fixed effects model was used only when I²<50% and P>0.10 for the Q test. When heterogeneity was large, its sources were explored based on subgroup analysis by diabetes (present or absent) and HF type (HFrEF or HFpEF). Sensitivity analysis for stability assessment of the results was conducted. Univariate meta-regression analysis was performed to find the source of heterogeneity. Publication bias for the primary outcome measure was assessed and quantified by the Egger’s test and funnel plots. The study results were subjected to a dose-response MA to investigate the association of the TyG index with the outcomes. Generalized least squares (GLS) was used to estimate parameters (27). The median of the TyG index in each stratum was used as an exposure dose. If it was not mentioned in the literature, an approximate median could be estimated based on calculation of the mean of the upper and lower limits. For open intervals, it was estimated based on the range of adjacent intervals. The presence/absence of a nonlinear association was explored by fitting a nonlinear model. If P > 0.05, it indicated that there was no nonlinear association, and a linear model was therefore used for fitting data. P<0.05 indicated a statistically significant difference. Statistical analysis was performed by Stata 15.

## 3 Results

### 3.1 Characteristics of the studies included

**Figure 1:**
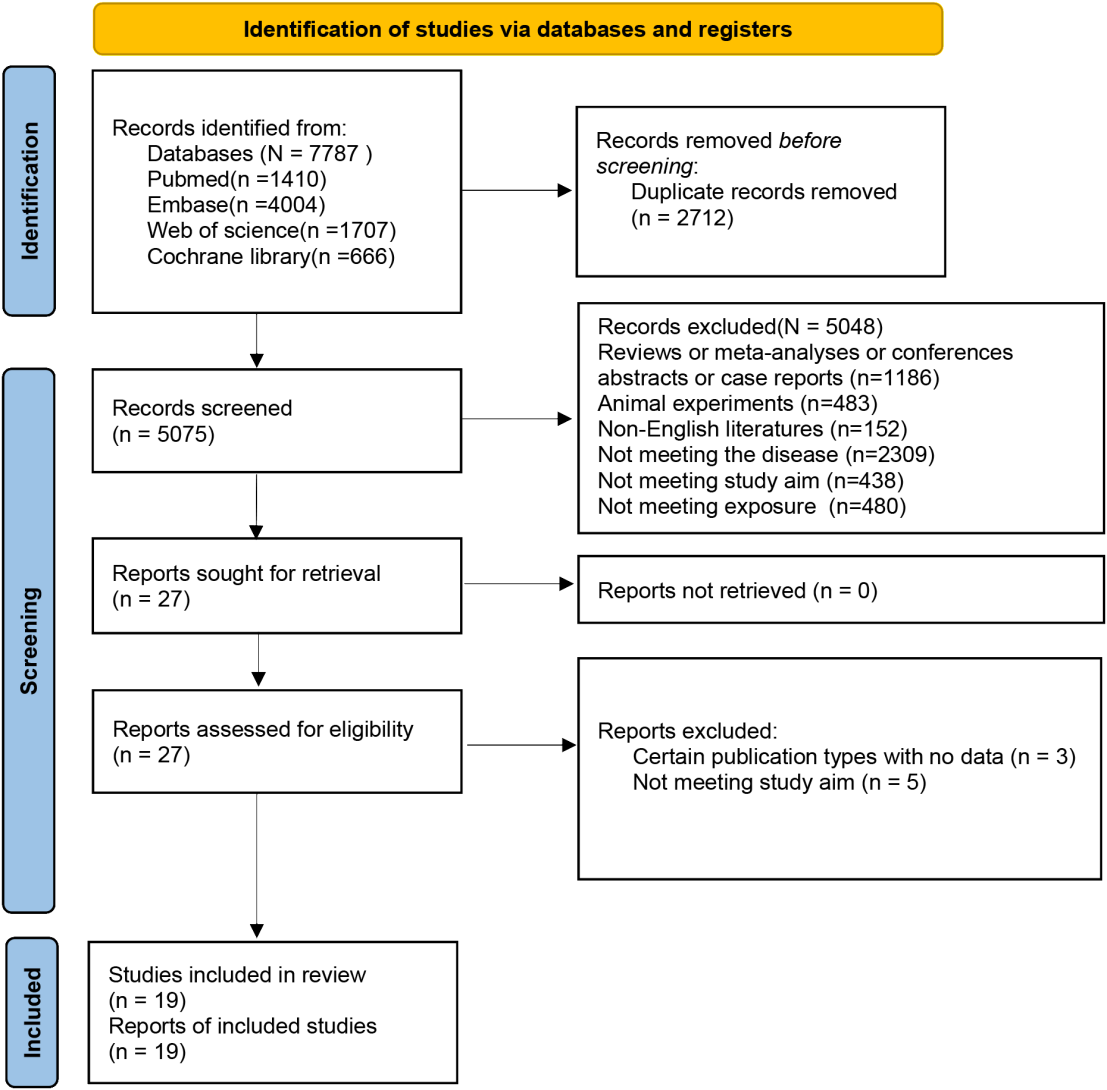
Process of study selection.

7787 studies were initially retrieved from the four databases. Of them, 2712 were excluded due to duplications. Subsequently, 5048 of them were deleted in the process of title/abstract screening, and eight of them deleted in full-text assessment for the reasons shown in **Figure 1**. In the end, this MA included nineteen studies (20–22, 24, 25, 28–41).

As shown in **Table 1**, the included studies involved 44275 patients in total. Published within the last 5 years, all these studies had a sample size ranging from 275 to 10780. The patients in the nineteen studies were aged 61.5-81 years on average. Male patients accounted for 44.7% -81.9%. The duration of follow-up was 12 months-7.33 years. Three studies included diabetic patients. Two studies included non-diabetic patients. In sixteen studies, there were diabetic and non-diabetic patients. **Table 1** presents an overall summary of the characteristics of each study, including its type, sample size, mean age, percentage of men, percentage of diabetic patients, LVEF, TyG index, follow-up time, and study outcome. The NOS was used to assess methodological quality. The results showed only one study awarded six stars and all the other studies awarded seven or more stars, indicating that they were all of good quality. Scores are detailed in **Supplementary File 2**.

**Table 1:** Main characteristics of the studies included.

Study	Year	Country	Sample size	Mean age	Male (%)	Diabetes (%)	LVEF (%)	TyG index group	Follow-Up (mean)	Outcome
Guo et al. (27)	2021	China	546	65.18 ± 12.01	66.3	100	40.22 ± 14.86	T1 (index value< 8.55) T2 (8.55 ≤ the index value < 9.06) T3 (index value≥9.06)	12 months	MACE; CV death
Han et al. (28)	2022	China	4411	70.6 ± 12.16	48.4	32.1	49.1 ± 10.4	T1 (index value< 8.25) T2 (8.25 ≤ the index value < 8.78) T3 (index value≥ 8.78)	NR	In-hospital mortality
Huang et al. (29)	2022	China	932	70.01 ± 2.96	62.1	32.8	≤40 (52.9%) 41~49 (16.5%) ≥ 50 (30.6%)	T1 (index value<8.83) T2 (8.83 ≤index value < 9.32) T3 (index value≥9.32)	478 days	All-cause death; CV death; MACEs
Özcan et al. (22)	2023	Turkey	773	61.5 ± 14.2	81.9	35.6	30 ± 7.43	T1 (index value: 5.3 [4.2–6.0]) T2 (index value: 8.7 [8.0–9.8]) T3 (index value: 15.3 [13.2–20.5]).	38 mouths	Long-term all-cause mortality
Sun et al. (30)	2023	China	2055	60.3 ± 11.0	82.2	38.5	40.6 ± 6.2	Q1 (index value< 8.54) Q2 (8.54 ≤index value < 8.93) Q3 (8.93 ≤index value< 9.41) Q4 (index value ≥ 9.41)	36 mouths	MACEs; all-cause mortalit
Zhou Q et al. (31)	2023	China	823	73.0 ± 12.7	48.1	42	64.6 ± 7.7	T1 (index value ≤ 8.47) T2 (8.48 ≤index value < 8.98) T3 (index value>8.98)	3.16 years	ACD; CV death
Zhou Y et al. (32)	2023	China	6697	63.3 ± 14.2	68.37	44.6	≤40 (33.16%) 41–49 (21.40%) ≥50 (45.44%)	T1 (index value < 8.40) T2 (8.40 ≤index value < 8.93) T3 (index value≥8.93)	3.9 years	ACD; CV death
Cheng et al. (20)	2024	China	886	68.90 ± 14.85	55.5	0	43.30 ± 10.40	D1 (index value ≤ 9.44) D2 (index value> 9.44)	NR	In-hospital mortality
Su et al. (21)	2024	China	717	>60 (50.21%) ≤60 (49.79%)	60.81	34.59	≤ 30	low-TyG (index value <8.48) high-TyG (index value ≥8.48)	21.58 mouths	MACEs
								low-TyG (index value<8.50) high-TyG (index value≥8.50)		All-cause mortality
Iwakura et al. (33)	2023	Japan	917	81 ± 9	44.7	39.3	>50	Q1 (7.26 ≤index value≤ 8.15) Q2 (8.16 ≤index value ≤ 8.50) Q3 (8.51 ≤index value≤ 8.87) Q4 (8.88≤index value ≤ 10.60)	387 days	all-cause death; MACE
Cunha et al. (34)	2021	Portugal	275	69 ± 13	70.5	NR	<30(58.2%) ≤40 (21.4%) 40–49 (16.5%) ≥50 (3.9%)	Median 8.65 (8.27-9.13).	5 years	All-cause mortality
Chen et al. (35)	2024	Israel	10780	68.11 ± 12.87	62.42	37.8	NR	Q1: 7.75 ≤index value < 8.71; Q2: 8.71 ≤index value < 9.13; Q3: 9.13 ≤index value < 9.57; Q4: 9.57 ≤index value < 10.70	1 year	1 year mortality
Jia et al. (36)	2024	China	1247	71.0 ± 14.2	61.1	34	≤40 (30.5%) 40–50 (23.7%) >50 (45.8%)	Q1, TyG index < 8.08; Q2,8.08 ≤index value < 8.51; Q3, 8.51≤index value ≤ 9.03; Q4, index value > 9.03	1079 days	all-cause mortality
Lai et al. (37)	2025	China	466	74 ± 10.41	60.09	100	61.51 ± 7.21	Q1 (7.3≤index value < 8.4) Q2 (8.4≤index value < 8.9) Q3 (8.9≤index value < 9.4) Q4 (9.4≤index value < 10.9)	7.33 years	Overall mortality
Liu et al. (39)	2024	China	1184	77.64 ± 9.37	47.97	100	62.80 ± 8.23	low-stable group (index value <8.26) medium-stable group (8.26≤index value < 9.06) high-increasing group (index value≥9.06)	29 months	MACEs; Cardiovascular death
Ni et al. (38)	2025	China	8693	70.59 ± 10.6	58.04	46.47	64.03 ± 7.91	TyG index was categorized into quartiles	2.56 years	MACE
Nishigoori et al. (24)	2024	Japan	892	NR	NR	NR	NR	Q1 index value ≤ 8.493 Q2/Q3 8.494≤index value< 9.536 Q4 index value > 9.537	365 days	30-day mortality
Zhang et al. (25)	2025	China	652	66.20 ± 14.86	70.6	0	36.35 ± 9.66	Tertile 1 index value < 8.19 Tertile 2 8.19≤index value< 8.55 Tertile 3 index value ≥ 8.55	1 year	All-cause mortality; CVD mortality
Zhou Z et al (40).	2024	Israel	1329	71.2 ± 12.9	55.8	26.6	NR	T1 (index value ≤ 9.03) T2 (9.03<index value ≤ 9.45) T3 (9.45<index value ≤ 9.85) T4 (index value >9.85)	3.9 years	5-year mortality

LVEF, Left Ventricular Ejection Fractions; NR, Not report.

### 3.2 Primary outcome measure

**Figure 2:**
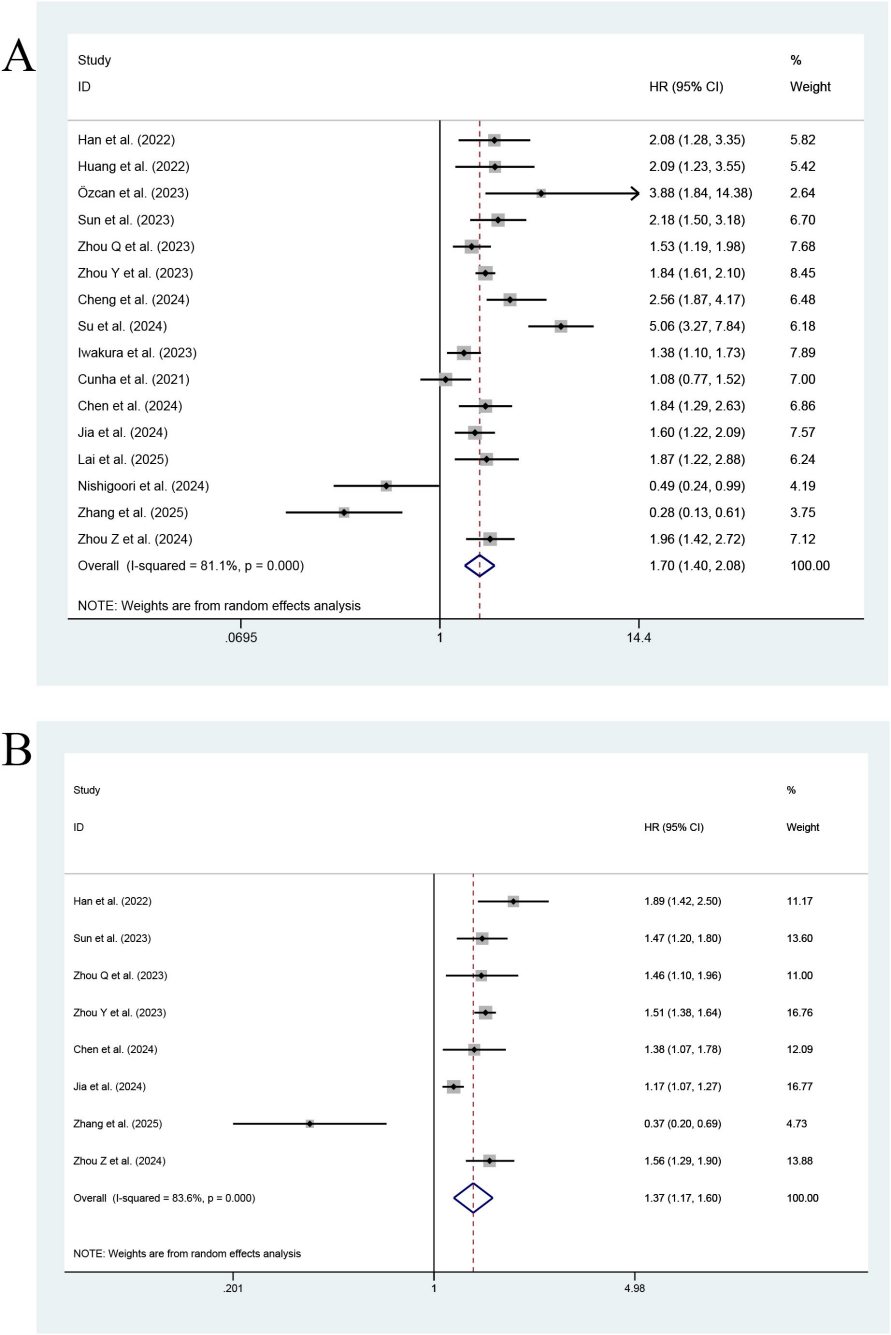
Forest plot of the effect of this index on ACD [**(A)** Categorical variables; **(B)** Continuous variables].

**Figure 2** shows that in an MA of the TyG index assessed as a categorical variable, a total of sixteen studies reported its effect on ACD in HF patients. Such patients with a high index value were at a significantly higher risk of ACD than those with a low index value, and the difference was statistically significant (HR=1.70; 95% CI: 1.40-2.08, P < 0.001). At the same time, heterogeneity among the studies was significant (I^2^ = 81.1%, p<0.001). In an MA of the TyG index (a continuous variable (CV)), a total of eight studies reported TyG. Each unit increase in the TyG index was associated with a 1.37-fold increase in the incidence of ACD in HF patients (95% CI: 1.17-1.60, P<0.001). Meanwhile, significant heterogeneity was found among the studies (I^2^ = 83.6%, P<0.001).

### 3.3 Secondary outcome measures

**Figure 3:**
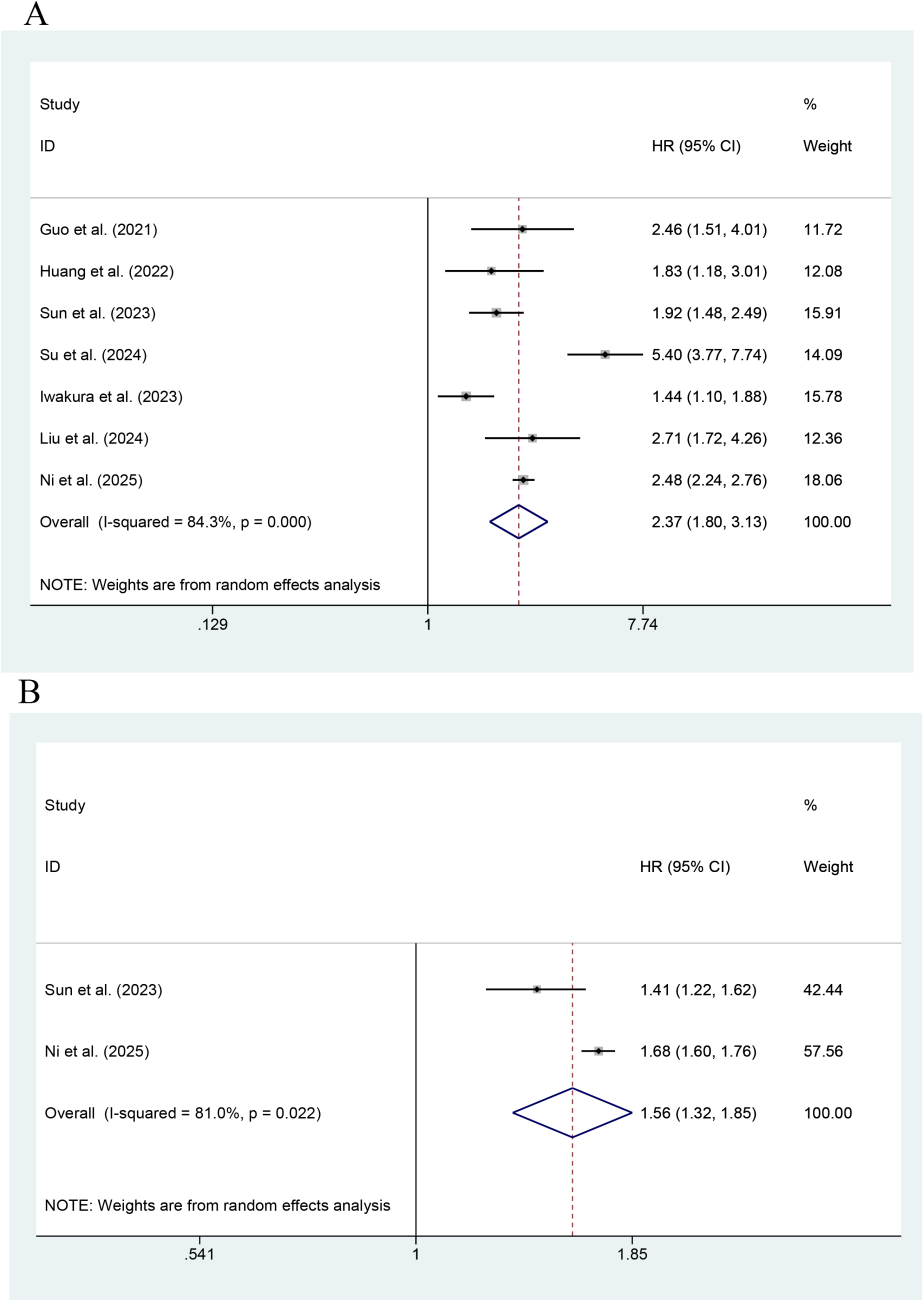
Forest plot of the effect of this index on MACEs [**(A)** Categorical variables; **(B)** Continuous variables].

**Figure 3** shows that seven studies reported the effect of the TyG index on MACEs. The highest TyG index had an association with an increase in the risk of MACEs, as compared with the lowest TyG index (HR=2.37; 95% CI: 1.80-3.13, P < 0.001, indicating a statistically significant difference). Heterogeneity among the studies was significant (I^2^ = 84.3%, P<0.001). The pooled results of two studies showed that the risk of MACEs increased with every unit increase in the TyG index as a continuous variable (HR=1.56; 95% CI: 1.32-1.85, P<0.001).

**Figure 4:**
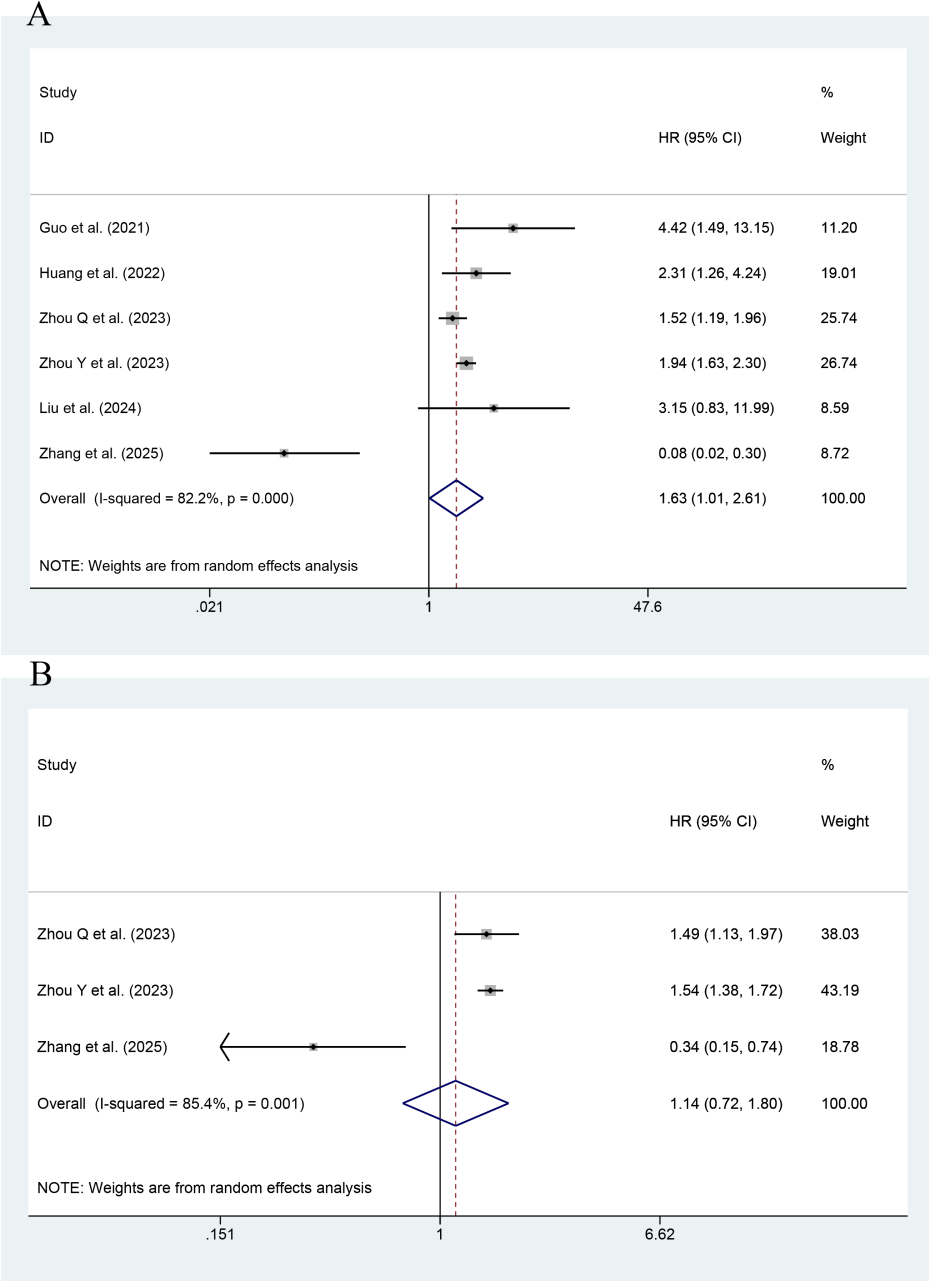
Forest plot of the effect of this index on CV death [**(A)** Categorical variables; **(B)** Continuous variables].

As shown in **Figure 4**, the effect of the TyG index on CV death was reported in six studies. The highest value of the TyG index had an association with an increase in the risk of CV death, as compared with its lowest value (HR=1.63; 95% CI: 1.01-2.61, P<0.001). Low heterogeneity occurred among the studies (I^2^ = 82.2%, P< 0.001). As shown by an MA of this index as a continuous variable, the risk of CV death was reported to increase with every unit increase in this index in a total of three studies (HR=1.14; 95% CI: 0.72-1.80, P=0.566). The results were not statistically significant.

### 3.4 Subgroup analyses

A subgroup analysis showed a significant association of this index with an increased risk of ACD in patients with HF complicated by reduced ejection fraction (HFrEF) (HR=2.43; 95% CI: 1.43-4.13; P=0.001). For patients with HF complicated by preserved ejection fraction (HFpEF), the HR for ACD was 1.85 (95% CI: 1.19-2.86; P=0.006). In diabetic patients, this index was associated with the risk of ACD (HR=1.45, 95% CI: 1.21-1.74; P<0.001). In non-diabetic patients, the index was not associated with the risk of ACD (HR=1.34, 95% CI: 0.85-2.12; P=0.209).

A subgroup analysis showed an association of the TyG index with the risk of CV death in HFrEF patients (HR=1.32, 95% CI: 1.06-1.65; P=0.014). However, for HFpEF patients, such an association was not statistically significant (HR=2.43, 95% CI: 0.96-6.18; P=0.061). In diabetic patients, the association was significant (HR=1.98, 95% CI: 1.31-3.01; P=0.001). In non-diabetic patients, the association was not statistically significant (HR=0.80, 95% CI: 0.22-2.89; P=0.735).

A subgroup analysis showed that in diabetic patients, the TyG index was associated with the occurrence of MACEs (HR=2.04, 95% CI: 1.39-2.99; P<0.001). In non-diabetic patients, the TyG index had an association with the risk of MACEs (HR=2.13, 95% CI: 1.14-3.99; P=0.017).

The above analyses showed that this index had different prognostic values in patients with different HF types (HFrEF and HFpEF) and diabetic status (**Table 2**).

**Table 2:** Results from subgroup analyses of outcome measures.

Outcome	Subgroups	No. of studies	HR (95%CI)	P-value	Heterogeneity within subgroup
All-cause death	HF type				
	HFrEF	5	2.43 (1.43, 4.13)	P=0.001	I^2^ = 90.7% P<0.001
	HFpEF	4	1.85 (1.19, 2.86)	P=0.006	I^2^ = 89.8% P<0.001
	Diabetes				
	Present	5	1.45 (1.21, 1.74)	P<0.001	I^2^ = 27.3% P=0.240
	Absent	6	1.34 (0.85, 2.12)	P=0.209	I^2^ = 88.8% P<0.001
CV death	HF type				
	HFrEF	1	1.32 (1.06, 1.65)	P=0.014	
	HFpEF	2	2.43 (0.96, 6.18)	P=0.061	I^2^ = 95.6% P<0.001
	Diabetes				
	Present	4	1.98 (1.31, 3.01)	P=0.001	I^2^ = 12.4% P=0.331
	Absent	3	0.80 (0.22, 2.89)	P=0.735	I^2^ = 91.2% P<0.001
MACEs	HF type				
	HFrEF	2	3.20 (1.16, 8.81)	P=0.025	I^2^ = 95.2% P<0.001
	HFpEF	2	1.92 (1.13, 3.26)	P=0.016	I^2^ = 92.7% P<0.001
	Diabetes				
	Present	4	2.04 (1.39, 2.99)	P<0.001	I^2^ = 61.4% P=0.051
	Absent	2	2.13 (1.14, 3.99)	P=0.017	I^2^ = 82.3% P=0.018

### 3.5 Meta-regression

Meta-regression analysis was performed to identify the sources of heterogeneity and explore the impact of potential variables associated with HF prognosis. The results showed that year of study publication was negatively associated with the effect size of CV death (P = 0.006), and the percentage of diabetic people was weakly positively associated with the effect size of CV death (P = 0.013). No significant differences were found in study design, sample size, age, gender, or LVEF. However, heterogeneity in all-cause death and MACEs remains unexplained. The results are detailed in **Table 3**.

**Table 3:** Meta-regression.

Variable	Meta-regression coefficient	95%CI	p
All-cause mortality			
Year of publication	-0.076189	-.3644366 to 0.2120569	0.604
Design (retrospective vs. prospective)	0.44688	-0.184955 to 1.078731	0.166
country (china vs. others)	0.2515201	-0.3478872 to 0.8509273	0.411
Sample size	0.0000237	-0.0000814 to 0.0001289	0.658
Age	-0.0130118	-0.0506459 to 0.0246223	0.498
Male sex	-0.0010449	-0.0269456 to 0.0248558	0.937
LVEF	0.0031624	-0.0490644 to 0.0553892	0.906
diabetes	0.0049937	-0.0077672 to 0.0177547	0.443
MACE			
Year of publication	0.1136083	-0.1484233 to 0.37564	0.395
Design (retrospective vs. prospective)	0.5937599	-0.1853054 to 1.372825	0.135
country (china vs. others)	0.5937599	-0.1853054 to 1.372825	0.135
Sample size	-0.000000803	-0.000121 to 0.0001194	0.990
Age	-0.0085897	-0.0387004 to 0.021521	0.576
Male sex	0.0020284	-0.0273963 to 0.0314531	0.893
LVEF	0.0086657	-0.0020366 to 0.019368	0.113
diabetes	0.0013676	-0.0113123 to 0.0140475	0.833
CV mortality			
Year of publication	-0.743868	-1.276873 to -0.2108636	0.006
Design (retrospective vs. prospective)	0.2336475	-0.0634507 to 0.5307458	0.123
Sample size	0.0000361	-0.0000114 to 0.0000837	0.136
Age	-0.0180195	-0.0478146 to 0.0117756	0.236
Male sex	0.0080474	-0.0066332 to 0.0227279	0.283
LVEF	0.0592551	-0.0880227 to 0.2065329	0.430
diabetes	0.027443	0.0058636 to 0.0490223	0.013

### 3.6 Dose–response relationship

**Figure 5:**
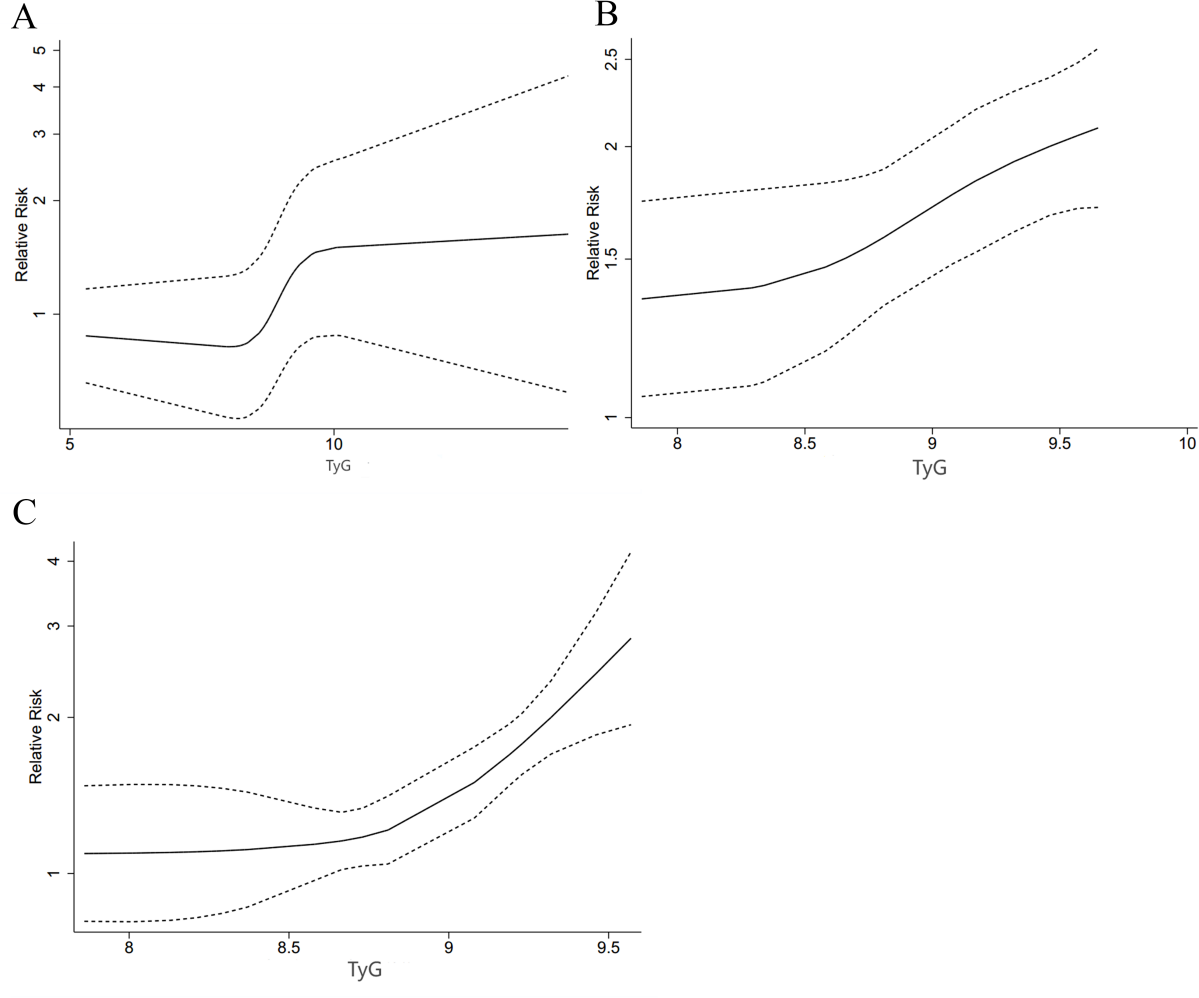
DRR between this index and HF outcome measures [**(A)** ACD; **(B)** MACEs; **(C)** CV death].

A restricted cubic spline (RCS) was used to look into the nonlinear link. A nonlinear DRR between the TyG index and ACD was found (P for nonlinearity < 0.001, **Figure 5A**). As shown in the figure, the risk of ACD decreased and then increased with the increase of TyG when the TyG index was less than 10. When the TyG index was more than 10, HRs tended to be stable.

There was also a nonlinear DRR between this index and the incidence of MACEs (P for nonlinearity = 0.001, **Figure 5B**). The figure shows that the HR for MACEs increased slowly when the TyG index was less than 8.5. The HR exhibited a greater upward trend when the TyG index was more than 8.5.

There was also a nonlinear DRR with CV death (P for nonlinearity < 0.001, **Figure 5C**). The HR for CV death increased more significantly as the TyG index increased.

### 3.7 Sensitivity analyses and publication bias

**Figure 6:**
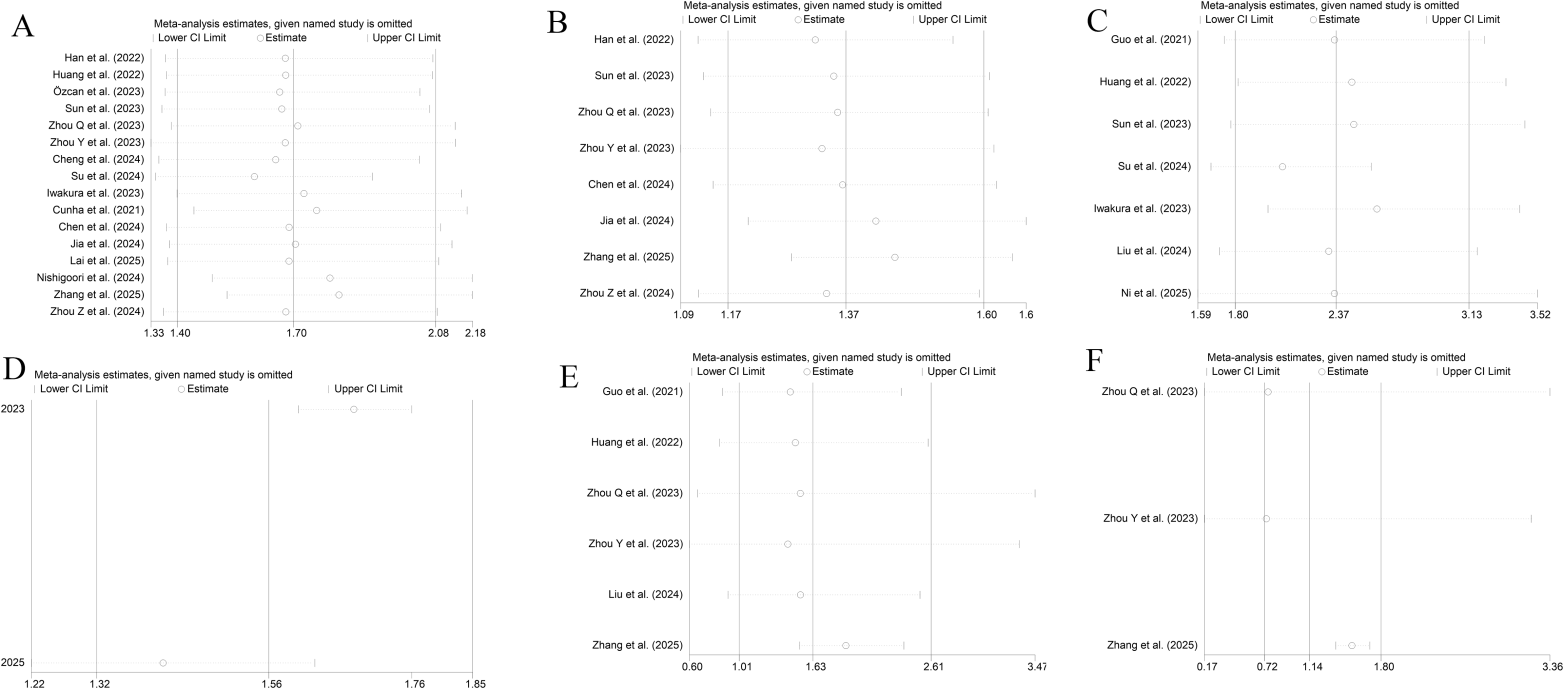
Sensitivity analyses [**(A, B)** All-cause death; **(C, D)** MACEs; **(E, F)** CV death; **(A, C, E)** Categorical variables; **(B, D, F)** Continuous variables].

As shown in **Figure 6**, the TyG index as an either categorical or continuous variable exhibited good stability in the sensitivity analyses of ACD, MACEs, and CV death.

A sensitivity analysis of the dose-response relationship was also performed. The results showed that the nonlinear dose-response relationships with ACD and CV death were robust. After elimination of individual studies one by one, the nonlinear associations with ACD and CV death remained significant (P for nonlinearity < 0.001). However, after elimination of the study by Sun, et al.[30], the dose-response relationship with MACEs shifted from nonlinear (P for nonlinearity < 0.001) to linear (P for nonlinearity = 0.62), with each 1-unit increase in the TyG index associated with an increased risk of MACEs (RR = 1.08, 95% CI: 1.05 -1.11) (**Supplementary File 3**). This suggests that the dose-response relationship with CV death may be influenced by specific studies and needs to be interpreted with caution.

**Figure 7:**
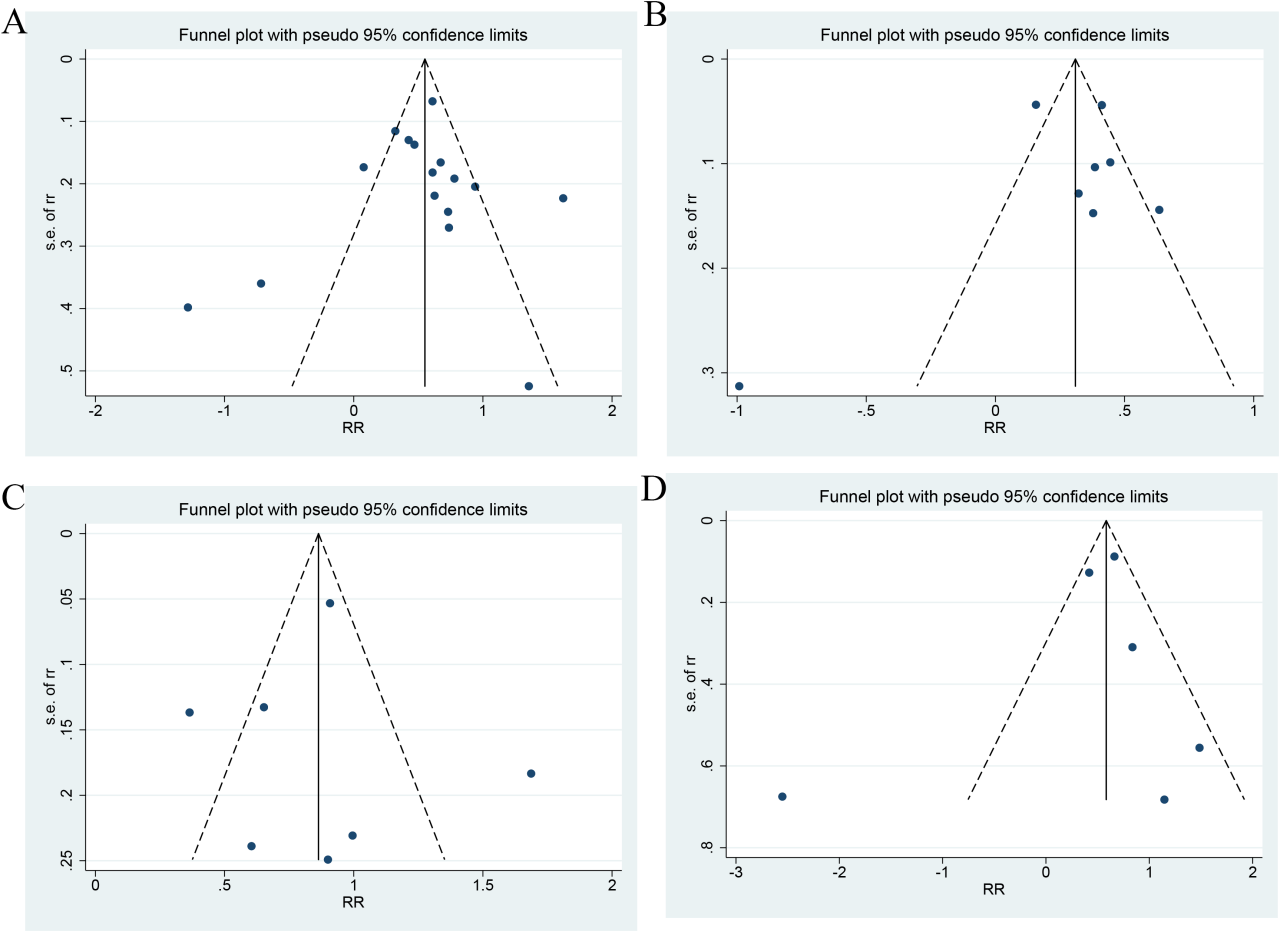
Publication bias (**(A)** Categorical variables of all-cause death; **(B)** Continuous variables of All-cause death; **(C)** Categorical variables of MACEs; **(D)** Categorical variables of CV death).

As shown in **Figure 7**, the Egger’s test showed no PB for ACD (P=0.775 for a categorical variable and P=0.919 for a continuous variable), MACEs (P=0.946), and CV death (P=0.686). Only two and three studies investigated the links of the TyG index as a continuous variable to MACEs and CV death, respectively. Therefore, no test for PB was performed.

## 4 Discussion

This MA is the first to investigate the association of this index with the prognosis in HF patients. It included nineteen studies involving 44275 HF patients. The results showed an association of the increase in the TyG index with an increased risk of ACD, MACEs, and CV death, and an association of the higher value of the TyG index with a greater risk of poor prognosis, regardless of the presence/absence of diabetes or any type of HF.

In addition, each unit increase in this index had an association with a 1.37-fold increase in the risk of ACD in HF. In this study, patients with the highest TyG index had a 2.37-fold and 1.63-fold higher risk of MACEs and CV death, respectively, as compared with those with the lowest value of this index. In non-diabetic patients with HF, a high TyG index was more significantly associated with the risk of ACD. In addition, a high value of this index was more significantly associated with the risk of ACD in patients with HFrEF. The MA results also showed a DRR. There was a nonlinear DRR between the TyG index and the incidence of ACD. When the TyG index was less than 10, the HR decreased and then increased with an increase in TyG. When the TyG index was more than 10, the HR changed little. A nonlinear DRR was observed between this index and the incidence of MACEs. When the TyG index was less than 8.5, there was a small increase in the HR. When the TyG index was more than 8.5, the HR changed greatly. The DRR between the TyG index and the incidence of CV death indicated that the HR exhibited a small to large change as the TyG index increased. The aim of this study was to quantify the stratification value of the TyG index for HF prognosis, rather than to verify their association alone. Despite differences in thresholds for grouping in the included studies, comparability of their results was enhanced by standardized HR calculations (the highest TyG index vs. the lowest TyG index) and dose-response analysis.

The results of this MA were consistent with the findings from a previous study, which indicated a positive association of the TyG index with the risk of poor prognosis in HF patients (42). This may be due to the fact that a high value of the TyG index reflects a high degree of IR, which in turn exacerbated metabolic disturbances and the inflammatory state, thereby aggravating HF patients’ condition. It has been shown that IR may result in an increased inflammatory response in the body (8). This inflammatory state will cause cardiovascular injuries (43). Inflammatory factors (e.g., CRP, TNF-α, and IL-6) will all be increased in IR state, and they can cause a direct injury to cardiomyocytes, thereby reducing myocardial function and exacerbating HF (8, 44, 45) IR may lead to endothelial dysfunction and increased permeability of the vascular wall, promote platelet aggregation and vasoconstriction, and ultimately result in vascular dysfunction and formation of atherosclerotic plaques (46). All these consequences pose a burden on the heart and further aggravate HF. IR will also affect the function of the autonomic nervous system (ANS), and especially activate the sympathetic nervous system (SNS) (44). Excessive sympathetic activity will lead to increased cardiac load, accelerate the heart rate, and increase myocardial oxygen consumption, which needs to be avoided especially in HF patients (47). In addition, prolonged sympathetic activation will lead to myocardial fibrosis (48), thereby further reducing the function of the heart to pump blood. IR may cause glucose metabolism disturbances, thereby resulting in hyperglycemia. Hyperglycemia and IR will lead to a range of metabolic disorders, including abnormal lipid metabolism (49). This will lead to increased low-density lipoprotein (LDL) and triglyceride, and decreased how-density lipoprotein (HDL) in blood (50), thereby promoting the occurrence of atherosclerosis and further aggravating HF. IR causes myocardial cells to fail to effectively utilize glucose and rely on fatty acid oxidation for energy supply. Fatty acid oxidation will increase oxygen demand by cardiomyocytes. In HF, there is insufficient oxygen supply to the heart, and this metabolic state may make cardiomyocytes more susceptible to injury (8). In summary, worsening of HF patients’ condition due to IR is mainly manifested through the mechanisms such as inflammatory response, endothelial dysfunction, autonomic imbalance, metabolic abnormalities, and lipid metabolism disorders. Therefore, this index is a marker of the severity of IR in patients. It plays an important role in prognostic assessment of HF.

The important role of diabetes and prediabetes in the development of HF have been reported previously (7, 51, 52). An MA including 28,643 participants showed that prediabetes had an association with a poorer prognosis in HF patients (52). In another meta-analysis including 129 studies, prediabetes was shown to have an association with poor prognosis of cardiovascular disease (53). Prediabetes and diabetes may cause changes in heart function, thereby increasing the risk of HF (54). IR has long been considered a major pathophysiological basis of diabetes. As a marker of IR, the TyG index was correlated with the development of antecedent diabetes and diabetes (55, 56). Therefore, the TyG index has a significant association with the prognosis of HF.

In addition, factors such as the severity of the HF patient’s condition and comorbidities may also affect such association. The predictive value of this index for the outcome of HF was also shown by our subgroup analyses to vary among different types of HF and among those with or without diabetes. The included studies also showed different prognostic predictive values of this index for HF due to different demographic characteristics such as age, gender, and BMI. However, the number of such studies was small, and valid data could not be extracted. It was therefore not possible to perform a systematic review.

This study clarified the association of the TyG index with the prognosis of HF and found its different predictive values in different subgroups. One of major findings in this study may be the clinical significance of the TyG index for HF patient management. This easy-to-measure index can benefit hospitals or clinics in resource-limited countries and settings, and it facilitates the stratification of the risk of HF patients, thereby ultimately providing better care and advice to patients. However, the cut-off value for the TyG index in current studies varies widely and lacks a uniform criterion, with most studies using 8.5-9.0 as a high TyG threshold, which may be influenced by population characteristics. We recommend that thresholds (e.g., TyG ≥ 8.5 in non-diabetic patients) be selected based on patient characteristics (e.g., diabetic status) in clinical practice. In the future, optimal cut-off values need to be determined based on large-scale cohorts and integrated into existing risk models (e.g., MAGGIC score) to improve predictive power.

However, this study has certain limitations. Firstly, except two studies from Turkey and Portugal, all the other studies were from Asian countries, including 13 from China. The results of this study may not be applicable globally but are still of some significance for predicting the prognosis of HF patients worldwide. Future validation of the prognostic value of the TyG index for the global population, especially people in Europe, North America, and Africa, is needed in a wider range of populations. Therefore, these studies were geographically restricted. This may lead to research bias. Secondly, differences in adjustments for confounders and inability to adequately adjust for them may result in biased estimates of associations. Thirdly, this MA focused only on the prognostic value (PV) of the TyG index in HF at baseline. There is still uncertainty about the impact of its longitudinal change on the risk of poor prognosis in HF patients. Therefore, the results of this MA should be interpreted with caution. It has been recently suggested (38) that TyG trajectories may have more powerful predictive value than single measurements, but the small number of studies did not allow for a meta-analysis. The PV of this index for adverse CV outcomes (ACVO) in HF patients should be validated by more large-scale prospective studies. Fourthly, there were some differences among the studies included. Some of them were possibly attributable to differences in the study population, design, sample size, and cut-off value applied. These differences may have a negative impact on the association involving TyG. Despite subgroup and regression analyses, only heterogeneity in CV death was found to possibly stem from the year of publication and the percentage of diabetic people, while no reasons were found for heterogeneity in all-cause death and MACEs. This may have an impact on the stability of the results. However, the results are still of some guiding significance for predicting the prognosis of HF in clinical practice. Fifthly, the use of different cut-off values in the studies was unavoidable, because they mostly categorized patients by tertile and quartile of this index. However, these tertiles and quartiles vary in different countries, settings, and populations. Therefore, no universal cut-off value of this index is available. These limitations have led to inconsistent findings. Sixthly, dose-response analyses in this study showed that the nonlinear associations with ACD and CV death were robust, but the association with MACEs may be sensitive to individual studies. For example, after elimination of the study by Sun, et al.[30], the dose-response relationship with MACEs shifted to linear, suggesting that the result was less stable and may be affected by between-study heterogeneity (e.g., differences in population characteristics or endpoint definitions). More high-quality studies are needed in the future to validate the dynamic association between MACEs and the TyG index. Finally, this MA was based on study-level data and could not adjust for confounders (e.g., medication use, comorbidity control) at the individual level, which may affect the precision of the risk estimates. Future studies should prioritize the collection of individual-level data to harmonize variable definitions and construct dynamic risk prediction models, so as to further validate the findings of this study.

## 5 Conclusion

In this MA, an increase in the TyG index was closely associated to an increase in the risk of ACD, MACEs, and CV death in HF patients. This MA suggests that clinicians should attach importance to this index in HF patients, especially non-diabetic patients and those with reduced ejection fraction. A high value of this index will portend a poorer prognosis. Measurement of the TyG index helps to identify the risk of HF patients and manage them appropriately. Therefore, timely monitoring and intervention of this index may help to improve the prognosis of HF patients. In addition, the results of this study provided an important insight for further exploration of the practical value of the TyG index for the prognosis of HF. A further study is required to investigate the impact of lifestyle changes (diet and exercise), pharmacologic interventions (PIs), and some parameters of other confounding factors (such as concomitant illnesses), so as to validate these findings.

## Data Availability

The original contributions presented in the study are included in the article/**Supplementary Material**. Further inquiries can be directed to the corresponding author.

## Supplementary material

Supplementary File 1 Record of search results.

Supplementary File 2 Quality assessment.

Supplementary File 3 Sensitivity of Dose-response analysis.
