# Supplementary figures and images for: A meta-analysis of the prognostic value of the TyG index in heart failure
Source: Front Endocrinol (Lausanne). 2025 Jul 24;16:1463647. doi: 10.3389/fendo.2025.1463647 (PMC12328152; doi:10.3389/fendo.2025.1463647)

Relative Risk

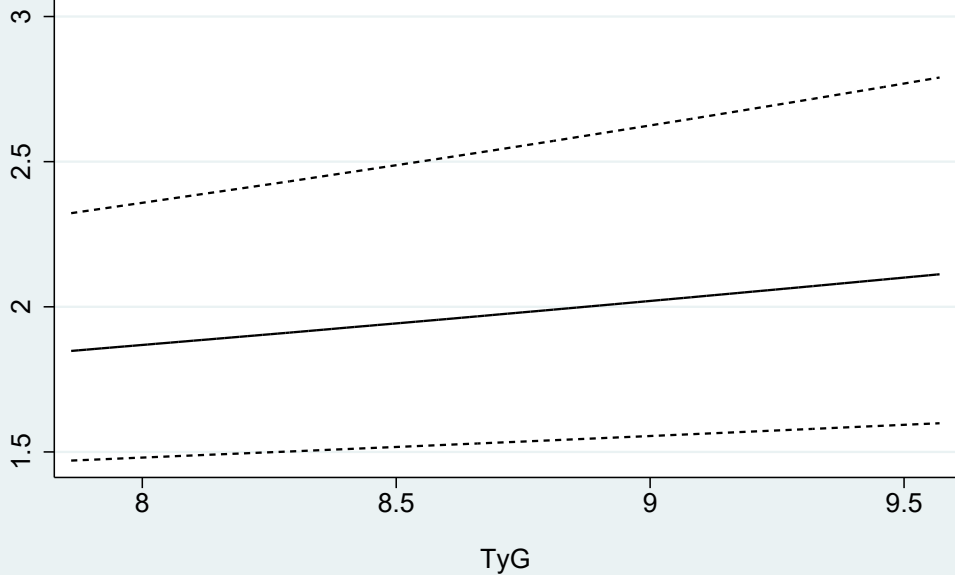

----- lclin

———— rr\_lin

----- ublin

Supplement: Supplementary File 1 — Record of search results. [file Image1.pdf]
