# Supplementary material for: A meta-analysis of the prognostic value of the TyG index in heart failure
Source: Front Endocrinol (Lausanne). 2025 Jul 24;16:1463647. doi: 10.3389/fendo.2025.1463647 (PMC12328152; doi:10.3389/fendo.2025.1463647)
Supplement: Supplementary File 2 — Quality assessment. [file Table1.docx]

Supplementary File 1 Record of search results

| PubMed | |
| --- | --- |
| # | Query |
| 1 | "glucose and triglycerides*"[Title/Abstract] OR "glucose-triglyceride* index"[Title/Abstract] OR "Triacylglycerol*"[Title/Abstract] OR "triglyceride and glucose index"[Title/Abstract] OR "Triglyceride*"[Title/Abstract] OR "triglyceride* x glucose*"[Title/Abstract] OR "triglyceride*-glucose index"[Title/Abstract] OR "TyG index"[Title/Abstract] |
| 2 | Triglycerides[MeSH Terms] |
| 3 | "cardia* decompensation"[Title/Abstract] OR "cardia* insufficiency"[Title/Abstract] OR "Cardiac Failure"[Title/Abstract] OR "cardiac incompetence"[Title/Abstract] OR "cardiac stand still"[Title/Abstract] OR "decompensatio cordis"[Title/Abstract] OR "Heart Decompensation"[Title/Abstract] OR "Heart Failure"[Title/Abstract] OR "heart incompetence"[Title/Abstract] OR "heart insufficiency"[Title/Abstract] OR "insufficientia cardis"[Title/Abstract] OR "Myocardial Failure"[Title/Abstract] OR "myocardial insufficiency"[Title/Abstract] |
| 4 | Heart Failure[MeSH Terms] |
| 5 | (#1 OR #2) AND (#3 OR #4) |

| Embase | |
| --- | --- |
| # | Query |
| 1 | 'triglyceride-glucose index'/exp |
| 2 | 'glucose and triglycerides*':ti,ab,kw OR 'glucose-triglyceride* index':ti,ab,kw OR 'triacylglycerol*':ti,ab,kw OR 'triglyceride and glucose index':ti,ab,kw OR 'triglyceride*':ti,ab,kw OR 'triglyceride* x glucose*':ti,ab,kw OR 'triglyceride*-glucose index':ti,ab,kw OR 'tyg index':ti,ab,kw |
| 3 | 'heart failure'/exp |
| 4 | 'cardia* decompensation':ti,ab,kw OR 'cardia* insufficiency':ti,ab,kw OR 'cardiac failure':ti,ab,kw OR 'cardiac incompetence':ti,ab,kw OR 'cardiac stand still':ti,ab,kw OR 'decompensatio cordis':ti,ab,kw OR 'heart decompensation':ti,ab,kw OR 'heart failure':ti,ab,kw OR 'heart incompetence':ti,ab,kw OR 'heart insufficiency':ti,ab,kw OR 'insufficientia cardis':ti,ab,kw OR 'myocardial failure':ti,ab,kw OR 'myocardial insufficiency':ti,ab,kw |
| 5 | (#1 OR #2) AND (#3 OR #4) |

| Cochrane library | |
| --- | --- |
| # | Query |
| 1 | (‘glucose and triglycerides*’ OR ‘glucose-triglyceride* index’ OR ‘Triacylglycerol*’ OR ‘triglyceride and glucose index’ OR ‘Triglyceride*’ OR ‘triglyceride* x glucose*’ OR ‘triglyceride* glucose index’ OR ‘TyG index’):ti,ab,kw |
| 2 | MeSH descriptor: [Triglycerides] explode all trees |
| 3 | (‘cardia* decompensation’ OR ‘cardia* insufficiency’ OR ‘Cardiac Failure’ OR ‘cardiac incompetence’ OR ‘cardiac stand still’ OR ‘decompensatio cordis’ OR ‘Heart Decompensation’ OR ‘Heart Failure’ OR ‘heart incompetence’ OR ‘heart insufficiency’ OR ‘insufficientia cardis’ OR ‘Myocardial Failure’ OR ‘myocardial insufficiency’ ):ti,ab,kw |
| 4 | MeSH descriptor: [Heart Failure] explode all trees |
| 5 | (#1 OR #2) AND (#3 OR #4) |

| Web of Science | |
| --- | --- |
| # | Query |
| 1 | glucose and triglycerides* (Topic) OR glucose triglyceride* index (Topic) OR triglyceride and glucose index (Topic) OR triglyceride* x glucose* (Topic) OR triglyceride* glucose index (Topic) OR TyG index (Topic) OR Triacylglycerol* (Topic) OR Triglyceride* (Topic) |
| 2 | Heart Failure (Topic) OR Heart Decompensation (Topic) OR heart incompetence (Topic) OR heart insufficiency (Topic) OR cardia* decompensation (Topic) OR cardia* insufficiency (Topic) OR Cardiac Failure (Topic) OR cardiac incompetence (Topic) OR cardiac stand still (Topic) OR decompensatio cordis (Topic) OR insufficientia cardis (Topic) OR Myocardial Failure (Topic) OR myocardial insufficiency (Topic) |
| 3 | #2 AND #1 |
